# The Therapeutic Efficacy and Safety of Compound Kushen Injection Combined with Transarterial Chemoembolization in Unresectable Hepatocellular Carcinoma: An Update Systematic Review and Meta-Analysis

**DOI:** 10.3389/fphar.2016.00070

**Published:** 2016-03-31

**Authors:** Xiao Ma, Rui-Sheng Li, Jian Wang, Yin-Qiu Huang, Peng-Yan Li, Ji Wang, Hai-Bin Su, Rui-Lin Wang, Ya-Ming Zhang, Hong-Hong Liu, Cong-En Zhang, Zhi-Jie Ma, Jia-Bo Wang, Yan-Ling Zhao, Xiao-He Xiao

**Affiliations:** ^1^Department of Pharmacy, 302 Military Hospital of People’s Liberation ArmyBeijing, China; ^2^Pharmacy College, Chengdu University of Traditional Chinese MedicineChengdu, China; ^3^Research and Technology Service Center, 302 Military Hospital of People’s Liberation ArmyBeijing, China; ^4^China Military Institute of Chinese Medicine, 302 Military Hospital of People’s Liberation ArmyBeijing, China; ^5^Department of Integrated Traditional Chinese and Western Medicine, West China Hospital, Sichuan UniversityChengdu, China; ^6^Liver Failure Treatment and Research Center, 302 Military Hospital of People’s Liberation ArmyBeijing, China; ^7^Department of Integrative Medical Center, 302 Military Hospital of People’s Liberation ArmyBeijing, China; ^8^Beijing Friendship Hospital, Capital Medical UniversityBeijing, China

**Keywords:** Compound Kushen Injection, transarterial chemoembolization, unresectable hepatocellular carcinoma, meta-analysis, systematic review

## Abstract

**Background:** Compound Kushen Injection (CKI) is a Chinese patent medicine approved by the China Food and Drug Administration for the treatment of various types of solid tumors. CKI, combined with transarterial chemoembolization (TACE), is believed to increase the therapeutic efficacy of unresectable hepatocellular carcinoma (HCC). We report an updated and extended meta-analysis with detailed outcomes of both the efficacy and adverse events (AEs) of CKI combined with TACE therapy.

**Materials and methods:** Electronic databases, including PubMed, Embase, the Cochrane Library, the Chinese Biomedical Database (CBM), Wanfang, the VIP medicine information system (VMIS) and the China National Knowledge Infrastructure (CNKI), were examined for relevant articles before November 13, 2015. An odds ratio (OR) was used to estimate tumor response (TR), Karnofsky Performance Scale (KPS) improvement, Child-Pugh (CP) improvement, survival rate (SR) and AEs. A publication bias and a subgroup analysis were also assessed.

**Results:** Eighteen studies, with a total of 1,338 HCC patients who met the criteria for the meta-analysis, were included. TR, KPS improvement and CP improvement were significantly enhanced for the combination therapy compared to TACE alone (OR = 1.84, 95% CI: [1.46, 2.33], *P* < 0.00001; OR = 2.37, 95% CI: [1.76, 3.18], *P* < 0.00001; OR = 1.81, 95% CI: [1.08, 3.03], *P* = 0.02, respectively). The combination therapy was associated with an improvement in 1-year and 2-year SRs but not an improved 3-year SR (OR = 2.40; 95% CI: [1.59, 3.62], *P* < 0.0001; OR = 2.49, 95% CI: [1.24, 5.00], *P* = 0.01; OR = 2.49, 95% CI: [0.94, 6.61], *P* = 0.07, respectively). A safety analysis indicated that AEs (including nausea/vomiting, fever, hepatalgia, increased transaminase, increased bilirubin and leukopenia) were reduced for the combination treatment compared to TACE alone.

**Conclusion:** The combination treatment of TACE and CKI was associated with improved TR, KPS and CP improvement and improved 1- and 2-year SRs in patients with unresectable HCC. The 3-year SR was not improved. The combination therapy resulted in a reduction in AEs. The findings of this study should be interpreted with caution because of the small sample size and study limitations.

## Introduction

Hepatocellular carcinoma is a health problem worldwide. More than 700,000 cases of HCC are diagnosed every year (Forner et al., 2012). HCC is the third cause of cancer-related deaths and the 16th absolute cause of death globally (Lozano et al., 2012). There has been a 62% increase in the number of HCC-related annual deaths over the last 20 years (from 463,000 to 752,000). The majority of HCC cases (80%) occur in sub-Saharan Africa and eastern Asia. The incidence rate is greater than 20 per 100,000 individuals (El-Serag, 2012; Llovet and Hernandez-Gea, 2014). The situation is particularly concerning in China. China accounts for 55% of HCC cases worldwide (Qin et al., 2013).

Surgery and liver transplant options are limited for HCC because only 30% of cases are diagnosed at an early stage that is amenable for resection, transplantation or local ablation with radiofrequency (European Association for the Study of The Liver and European Organisation For Research and Treatment Of Cancer, 2012). The remaining 70% are at an advanced stage with few therapeutic options. Transarterial chemoembolization is the current standard treatment for intermediate stage HCC. TACE significantly increases the SR in patients with unresectable HCC compared to supportive care or suboptimal therapies according several randomized trials and meta-analyses (Cammá et al., 2002; Llovet et al., 2002; Lo et al., 2002; Llovet and Bruix, 2003). However, approximately 50% of patients do not benefit from TACE (Lammer et al., 2010). A retrospective study demonstrated that 64% of patients received a second cTACE, but only a few patients (26%) received a third cTACE (Terzi et al., 2012). Several AEs (post embolization syndrome, hepatic insufficiency and myelosuppression) are associated with TACE therapy. The identification of new drugs that could be combined with TACE may enhance its therapeutic efficacy and reduce AEs.

**FIGURE 1:**
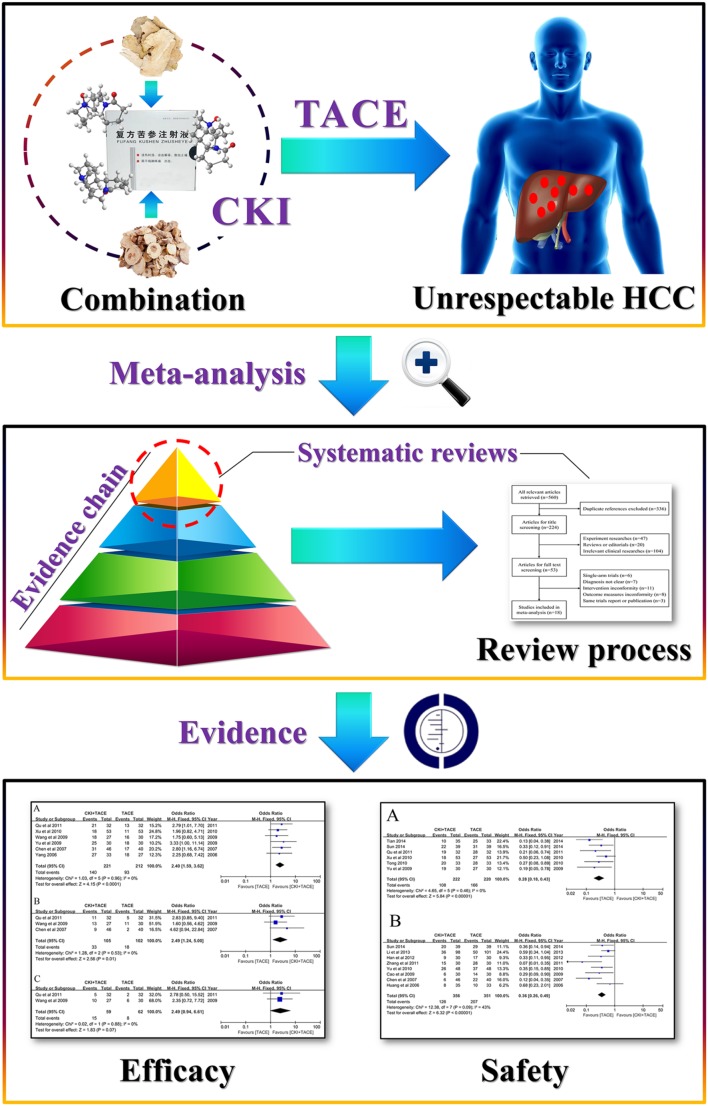
**Work flow of present study**.

Compound Kushen Injection is a Chinese patent medicine extracted from two medical herbs, *Radix Sophorae* Flavescentis and *Rhizoma Smilacis* Glabrae. It is approved by the China Food and Drug Administration (CFDA) for the treatment of various types of solid tumors. The main compounds in CKI are matrine, oxymatrine and sophoridine. These compounds are associated with liver protection and the treatment of viral hepatitis and HCC in China (Zhou et al., 2012; Wang et al., 2015; Parvez et al., 2016). The combination of CKI and TACE may increase the therapeutic efficacy of unresectable HCC treatment. A previous meta-analysis also confirmed that CKI plus TACE was superior to TACE alone in the treatment of unresectable HCC (TR, quality of life and 1-year SR) (Sun et al., 2011). We report an updated and extended meta-analysis with detailed outcomes for efficacy and AEs (**Figure 1**).

## Materials and Methods

### Literature Search Strategy

This meta-analysis was performed according to the PRISMA statement (Moher et al., 2009). Seven databases, including PubMed, Embase, the Cochrane Library, the CBM, Wanfang, the VMIS and the CNKI, were examined for original articles published before November 13, 2015. All searches were performed without language limitations to identify all relevant trials. The following initial search items were used: “HCC” [Title/Abstract] OR “hepatic tumor” [Title/Abstract] OR “liver cancer” [Title/Abstract] AND “CKI” [Title/Abstract] OR “Yan Shu Injection” [Title/Abstract]. The searched results were downloaded into Endnote software (version X7, Thomson Reuters, Inc., New York, NY, USA) for further screening.

### Review Strategy and Selection Criteria

The literature searches were performed using Endnote software. Duplicate records were deleted. Two investigators (Xiao Ma and Yin-Qiu Huang) independently performed the title/abstract review and the full-text review. Disagreements between the two investigators were resolved by consensus and discussion.

Studies that met the following inclusion criteria were considered eligible for this meta-analysis: (1) trials were described as RCTs; (2) study subjects or patients were confirmed cytologically or pathologically, or diagnosed by computed tomography (CT) with HCC; (3) patients in the experimental group received TACE-based therapy with CKI, whereas patients in the control group received TACE-based therapy only; and (4) a minimum of two of the following outcomes were included in each study: TR, KPS improvement, CP improvement, SR or AEs.

Trials were excluded if they did not meet the following criteria: (1) reviews, comments, case report, editorials, letters, or articles unrelated with the topics; (2) trials were not RCTs; (3) diagnostic criteria were not clearly reported in trials; and (4) the intervention of HCC patients was not based on TACE treatment.

### Data Extraction and Methodological Quality Assessment

Two independent investigators (Xiao Ma and Cong-En Zhang) extracted the following information from the selected studies: first author, year of publication, sample size regimens and outcome measures. If the identical trial appeared in different publications, reports with the most relevant information were discussed and included by investigators.

The methodological quality was evaluated according to the Cochrane Handbook for Systematic Reviews of Interventions (Deeks et al., 2011). The evaluation was performed as follows: selection bias, performance bias, detection bias, attrition bias, reporting bias and other biases. The quality judgment of each term was assessed using three levels: ‘Low risk’ of bias (adequate and correct description of methods or procedures), ‘High risk’ of bias (incorrect description of methods or procedures) or ‘Unclear risk’ of bias (no description of methods and procedures).

### Statistical Analysis

The analysis was conducted using Review Manager 5.3 (Cochrane Collaboration, Oxford, UK). Outcome measures, such as TR, KPS improvement, CP improvement, SR and AEs (**Supplementary Table S1**), were considered dichotomous variables. These parameters were expressed as an OR with 95% CI for each study. Heterogeneity among studies was estimated using the Cochran’s Q statistic and *I*^2^ tests. A *P*-value <0.1 or an *I*^2^ > 50% were defined to have heterogeneity (Higgins et al., 2003). A fixed-effects model was used to pool the estimates when heterogeneity was absent. A random-effects model was used to pool the estimates when there was obvious heterogeneity. Funnel plots were used to detect publication bias.

## Results

### Identification of Eligible Studies

A total of 560 potential articles were identified from the databases, and 336 articles were excluded after duplicate review. A total of 224 articles were excluded for title screening, and 171 articles were excluded because of irrelevant themes or experiments. Fifty three articles remained for full-text review.

**FIGURE 2:**
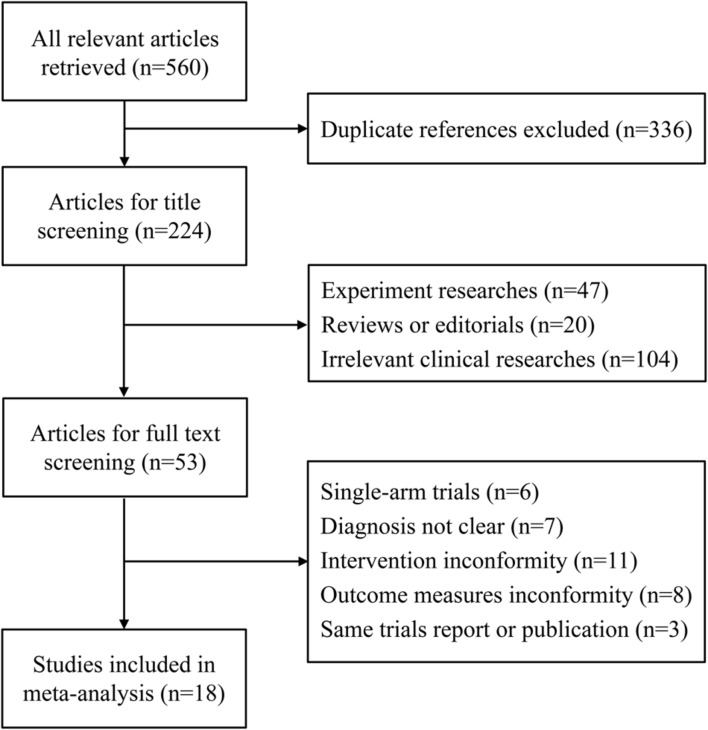
**Study selection process for the meta-analysis**.

In the review, 35 studies were excluded for the following reasons: 6 studies were single-arm designs, 7 studies were not clearly confirmed and diagnosed with HCC, 11 studies involved ambiguous therapies in the intervention group, 8 studies did not present definite outcome measures and 3 studies presented overlapping data with another study. Eighteen studies (Huang et al., 2006; Yang, 2006; Chen et al., 2007; Cao et al., 2009; Deng et al., 2009; Wang and Chen, 2009; Yu et al., 2009; Tong, 2010; Xu et al., 2010; Yu and Kang, 2010; Qu et al., 2011; Wang et al., 2011; Zhang and Tian, 2011; Zuo et al., 2011; Han et al., 2012; Li et al., 2013; Sun, 2014; Tian, 2014) that had a total of 1,338 HCC patients who met the criteria for the meta-analysis were included (**Figure 2**).

### Study Characteristics and Quality Assessment

**FIGURE 3:**
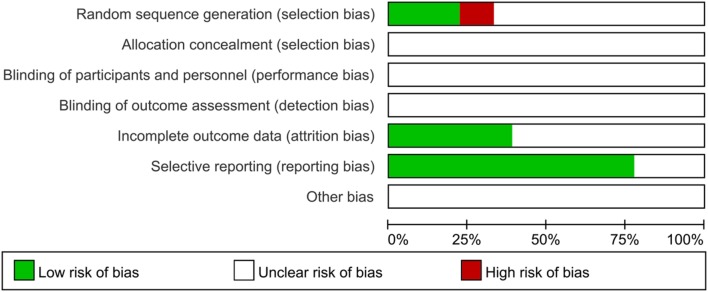
**Risk of bias assessment in the included trials.** The quality assessment was performed by Review Manager 5.3 according to the Cochrane Handbook for Systematic Reviews of Interventions, Version 5.1.0. The red square indicates a high risk of bias. The green square indicates a low risk of bias, and the blank square indicates an unclear risk of bias.

The baseline characteristics of the included studies are presented in **Supplementary Table S2**. Among the 18 studies (Huang et al., 2006; Yang, 2006; Chen et al., 2007; Cao et al., 2009; Deng et al., 2009; Wang and Chen, 2009; Yu et al., 2009; Tong, 2010; Xu et al., 2010; Yu and Kang, 2010; Qu et al., 2011; Wang et al., 2011; Zhang and Tian, 2011; Zuo et al., 2011; Han et al., 2012; Li et al., 2013; Sun, 2014; Tian, 2014) conducted between 2006 and 2014, all were RCTs with a comparison between a combination of CKI and TACE and TACE treatment alone. The age of the patients ranged from 25 to 79 years. Seventeen studies reported the stages of HCC and 13 mentioned KPS. The TACE treatment regimen varied greatly. However, the combination of 5-FU, DDP, MMC, EPI or DDP in the TACE treatment was the most common regimen. One study did NR the embolizing agents of TACE. Lipiodol was the embolizing agent in the remaining studies. The dose of administered CKI ranged from 15 to 30 mL/day. In the majority of studies, CKI was administered for 15 days via intravenous drip (**Table 1**). All trials mentioned “randomization,” but only four trials stated the appropriate generation of the random allocation sequence (Yang, 2006; Wang and Chen, 2009; Yu and Kang, 2010; Zuo et al., 2011). No trials described information on allocation concealment and blinding. Seven trials (Yang, 2006; Chen et al., 2007; Wang and Chen, 2009; Xu et al., 2010; Qu et al., 2011; Zuo et al., 2011; Li et al., 2013) mentioned over exclusion of participants or drop-outs. Selective reporting was low for the 14 trials (Yang, 2006; Chen et al., 2007; Cao et al., 2009; Wang and Chen, 2009; Yu et al., 2009; Tong, 2010; Xu et al., 2010; Yu and Kang, 2010; Qu et al., 2011; Zuo et al., 2011; Han et al., 2012; Li et al., 2013; Sun, 2014; Tian, 2014) (**Figure 3**, **Supplementary Figure S1**).

**Table 1:** Intervention characteristics of the included trials.

Study (years)	TACE treatment drugs	Embolizing Agents	CKI dosage and method	Duration	Outcome measures
Tian, 2014	5-FU + DDP + MMC	Lipiodol	20 mL/day, intravenous drip	6 weeks	TR, AEs
Zuo et al., 2011	5-FU + DDP + MMC	Lipiodol	20 mL/day, intravenous drip	14 days	TR, AEs
Sun, 2014	5-FU + DDP + MMC	Lipiodol	20 mL/day, intravenous drip	15 days/course, 3 courses	TR, KPS, AEs
Li et al., 2013	5-FU + DDP + MMC	Lipiodol	20 mL/day, intravenous drip	2 weeks/course, 2 courses	TR, KPS, CP, AEs
Han et al., 2012	EPI	Lipiodol	15 mL/day, intravenous drip	45 days	TR, KPS, CP, AEs
Zhang and Tian, 2011	5-FU + EPI + MMC	Lipiodol	20 mL/day, intravenous drip	20 days/course, 3 courses	TR, AEs
Wang et al., 2011	5-FU+EPI+MMC	Lipiodol	20 mL/day, intravenous drip	15 days/course, 2 courses	TR, KPS, AEs
Qu et al., 2011	CF+5-Fu+DDP	Lipiodol	20 mL/day, intravenous drip	14 days/course, 2 courses	KPS, SR, AEs
Tong, 2010	5-FU + DDP + EPI	Lipiodol	20 mL/day, intravenous drip	14 days/course, 2 courses	TR, AEs
Xu et al., 2010	CF + 5-Fu + DDP + THP or HCPT	Lipiodol	20 mL/day, intravenous drip	15 days/course, 2 courses	TR, KPS, SR, AEs
Yu and Kang, 2010	5-FU + ADM + HCPT	Lipiodol	20 mL/day, intravenous drip	15 days/course, 3 courses	TR, KPS, AEs
Cao et al., 2009	5-FU + DDP + MMC	Lipiodol	20 mL/day, intravenous drip	2 weeks	TR, KPS, AEs
Yu et al., 2009	5-FU + HCPT	NR	20 mL/day, intravenous drip	14 days/course, 2 courses	TR, SR, AEs
Deng et al., 2009	THP	Lipiodol	20 mL/day, intravenous drip	14 days/course, 2 courses	TR, KPS
Wang and Chen, 2009	5-FU + ADM + DDP	Lipiodol	20 mL/day, intravenous drip	10 days	TR, KPS, SR
Chen et al., 2007	5-FU + EPI + MMC	Lipiodol	20 mL/day, intravenous drip	2–3 weeks/course, 3 courses	TR, KPS, SR, AEs
Yang, 2006	5-FU + MMC + ADM	Lipiodol + GS	20 mL/day, intravenous drip	20 days	TR, KPS, SR
Huang et al., 2006	5-Fu + ADM + HCPT	Lipiodol + GS	15–30 mL/day, intravenous drip	10–20 days/course, 2–4 courses	TR, KPS, AEs


*CKI, Compound Kushen Injection; TACE, Transarterial chemoembolization; 5-FU, fluorouracil; DDP, cisplatin; MMC, mitomycin; EPI, epirubicin; CF, calcium folinate; THP, pirarubicin; HCPT, hydroxycamptothecin; ADM, adriamycin; NR, not report; GS, gelatin sponge; TR, tumor response; AEs, adverse events; KPS, Karnofsky Performance Scale; CP, Child-Pugh; SR, survival rate.*

### Tumor Response

**FIGURE 4:**
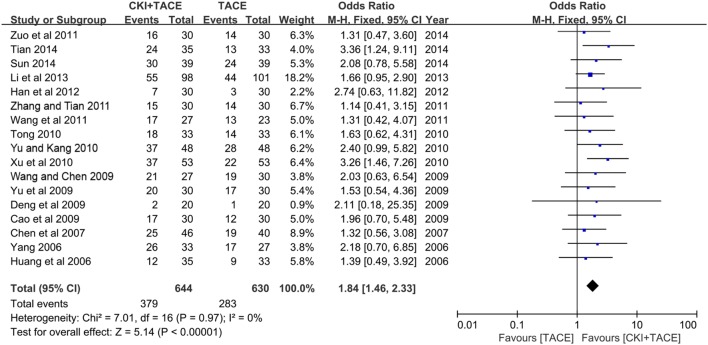
**Forest plot of the TR in patients treated with CKI + TACE therapy and TACE alone.** *I*^2^ and *P* are the criterion for the heterogeneity test, ♢ pooled OR, –□– OR and 95% CI.

Seventeen studies (Huang et al., 2006; Yang, 2006; Chen et al., 2007; Cao et al., 2009; Deng et al., 2009; Wang and Chen, 2009; Yu et al., 2009; Tong, 2010; Xu et al., 2010; Yu and Kang, 2010; Wang et al., 2011; Zhang and Tian, 2011; Zuo et al., 2011; Han et al., 2012; Li et al., 2013; Sun, 2014; Tian, 2014) provided the TRs (**Figure 4**). A meta-analysis of these studies using a fixed-effect model demonstrated that CKI combined with TACE therapy significantly improved the TR in the treatment of unresectable HCCs (OR = 1.84, 95% CI: 1.46, 2.33; *P* < 0.00001). No statistically significant heterogeneity (*P* = 0.97, *I*^2^ = 0%) was found among individual trials.

### KPS Improvement

**FIGURE 5:**
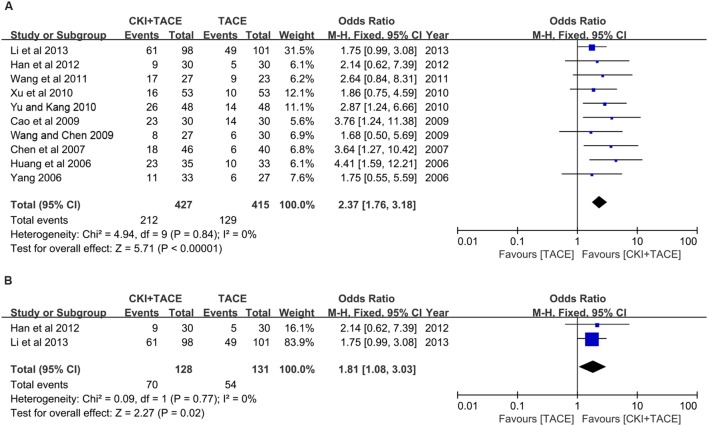
**Forest plot of KPS improvement and CP improvement in patients treated with CKI + TACE therapy and TACE alone.** **(A)** Forest plot of KPS improvement; **(B)** Forest plot of CP improvement. *I*^2^ and *P* are the criterion for the heterogeneity test, ♢ pooled OR, –□– OR and 95% CI.

There were 10 trials (Huang et al., 2006; Yang, 2006; Chen et al., 2007; Cao et al., 2009; Wang and Chen, 2009; Xu et al., 2010; Yu and Kang, 2010; Wang et al., 2011; Han et al., 2012; Li et al., 2013) that demonstrated a KPS improvement of >10 points. The pooled OR revealed that CKI combined with TACE therapy significantly enhanced KPS improvement compared to TACE alone (OR = 2.37, 95% CI: 1.76, 3.18; *P* < 0.00001). No significant heterogeneity (*P* = 0.84, *I*^2^ = 0%) was found among these trials (**Figure 5A**).

### Child-Pugh Improvement

Two trials (Han et al., 2012; Li et al., 2013) demonstrated CP improvement. The pooled analysis (using a fixed-effects model) suggested that CKI combined with TACE therapy significantly improved CP improvement compared to TACE alone in the treatment of unresectable HCC (OR = 1.81, 95% CI: 1.08, 3.03; *P* = 0.02). No statistically significant heterogeneity (*P* = 0.77, *I*^2^ = 0%) was found among individual trials (**Figure 5B**).

### Survival Rate

**FIGURE 6:**
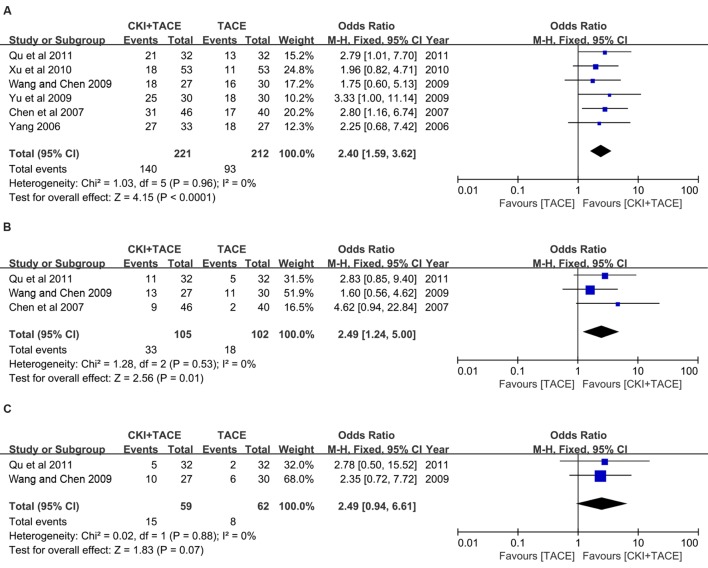
**Forest plot of the SR in patients treated with CKI + TACE therapy and TACE alone.** **(A)** Forest plot of the 1-year SR; **(B)** Forest plot of the 2-year SR; **(C)** Forest plot of the 3-year SR. *I*^2^ and *P* are the criterion for the heterogeneity test, ♢ pooled OR, –□– OR and 95% CI.

Six trials (Huang et al., 2006; Yang, 2006; Chen et al., 2007; Wang and Chen, 2009; Yu et al., 2009; Xu et al., 2010; Qu et al., 2011) with 433 patients were identified using the 1-year SR outcome. CKI combined with TACE therapy improved the 1-year SR in patients with unresectable HCC compared to TACE alone (OR = 2.40; 95% CI: 1.59, 3.62; *P* < 0.0001) (**Figure 6A**). No heterogeneity (*P* = 0.96, *I*^2^ = 0%) was noted among these studies. Three trials (Chen et al., 2007; Wang and Chen, 2009; Qu et al., 2011) reported the 2-year SR. A fixed-effect model demonstrated that CKI combined with TACE therapy significantly improved the 2-year SR (OR = 2.49, 95% CI: 1.24, 5.00; *P* = 0.01). No statistically significant heterogeneity (*P* = 0.53, *I*^2^ = 0%) was observed among individual trials (**Figure 6B**). There was no difference between CKI combined with TACE therapy and TACE treatment alone in 3-year SR (based on two studies) (Wang and Chen, 2009; Qu et al., 2011) (OR = 2.49, 95% CI: 0.94, 6.61; *P* = 0.07). No heterogeneity (*P* = 0.88, *I*^2^ = 0%) was noted. The fixed-effect model was used in the analysis (**Figure 6C**).

### Adverse Events

**Table 2** summarizes the forest plots for the AEs that occurred during the treatment. The non-hematological AEs included nausea/vomiting, fever, hepatalgia, increased transaminase and increased bilirubin (**Supplementary Figures S2A–F**). Leukopenia that was related to hematological AEs was recorded in eight trials (Huang et al., 2006; Chen et al., 2007; Cao et al., 2009; Yu and Kang, 2010; Zhang and Tian, 2011; Han et al., 2012; Li et al., 2013; Sun, 2014). No statistically significant heterogeneity was noted for the non-hematological AEs (*P*: 0.42–1.00; *I*^2^ = 0%). A single application of TACE alone resulted in a higher incidence of non-hematological AEs compared to CKI combined with TACE (*P* < 0.00001). CKI combined with TACE also demonstrated a lower rate of leukopenia (*P* < 0.00001). The heterogeneity is moderate (*P* = 0.09 and *I*^2^ = 43%).

**Table 2:** Comparison of AEs for patients treated with CKI+TACE therapy and TACE alone.

Adverse events	Trials	CKI + TACE	TACE	Heterogeneity		OR (95% CI)	*P*-value
						
				*P*	*I*^2^		
Nausea/Vomiting	11	94/374	173/366	1.00	0%	0.37 (0.27, 0.59)	<0.00001
Fever	8	140/287	217/283	0.67	0%	0.29 (0.20, 0.42)	<0.00001
Hepatalgia	6	102/216	159/212	0.42	0%	0.27 (0.17, 0.42)	<0.00001
Increased transaminase	8	131/295	204/283	0.52	0%	0.27 (0.18, 0.39)	<0.00001
Increased bilirubin	6	108/222	166/220	0.46	0%	0.28 (0.18, 0.43)	<0.00001
Leukopenia	8	126/356	207/351	0.09	43%	0.36 (0.26, 0.49)	<0.00001


**I*^2^ and *P* are the criterion for the heterogeneity test. The P-value indicates the significance between two arms.*

### Publication Bias

**FIGURE 7:**
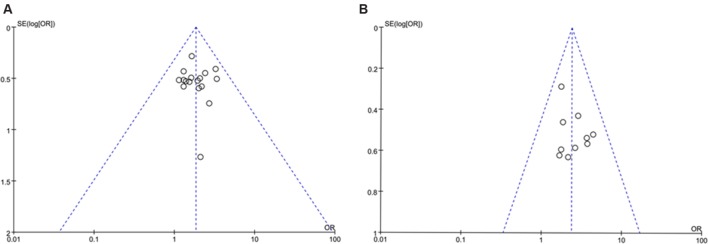
**Funnel plot for the publication bias.** **(A)** Funnel plot of the TR; **(B)** Funnel plot of KPS improvement.

A funnel plot was used to express the publication bias. There were 17 trials (Huang et al., 2006; Yang, 2006; Chen et al., 2007; Cao et al., 2009; Deng et al., 2009; Wang and Chen, 2009; Yu et al., 2009; Tong, 2010; Xu et al., 2010; Yu and Kang, 2010; Wang et al., 2011; Zhang and Tian, 2011; Zuo et al., 2011; Han et al., 2012; Li et al., 2013; Sun, 2014; Tian, 2014) included in the funnel plot of the TR, and no significant asymmetry was observed (**Figure 7A**). The funnel plot of KPS improvement was used to measure publication bias. The funnel plot indicated that there was no asymmetry in KPS improvement (**Figure 7B**).

## Discussion

Transarterial chemoembolization is one of the few effective therapeutic treatments for unresectable HCC. However, TACE only has a modest survival benefit (an average increase in survival of 3 months). Additionally, TACE is a potent stimulator of neo-angiogenesis (Cabrera et al., 2011). The observed angiogenesis upregulates local angiogenic factors, which can promote tumor growth. This growth increases the risk of metastases and poor outcomes (Sergio et al., 2008). Therefore, TACE is combined with a systemic anticancer agent, such as sorafenib. However, the efficacy and toxicity of this combination therapy is controversial (Park et al., 2012; Liu et al., 2014). A phase III study revealed that TACE in combination with sorafenib did not significantly prolong time to progression in Asian patients with HCC (Kudo et al., 2011). A new combination therapy for unresectable HCC patients in Asian countries is necessary.

Chinese practitioners have sought treatments for liver cancer for a long time. Traditional Chinese medicines are increasingly applied to enhance the efficacy of TACE and to reduce the side effects. A recent meta-analysis indicated that traditional Chinese medicines (used as an herbal formula) could increase the efficacy and reduce the toxicity of TACE in patients with unresectable HCC (Cheung et al., 2013). The Chinese patent medicine CKI, combined with TACE, was superior compared to TACE alone for unresectable HCC in TR, quality of life and 1-year survival (Sun et al., 2012). We further confirmed these results and report extended findings. CKI combined with TACE therapy was associated with a significantly higher TR, KPS improvement and CP improvement compared to TACE mono-therapy (*P* < 0.00001, *P* < 0.00001, *P* = 0.02, respectively). The combination therapy also improved the 1-year SR and the 2-year SR using the pooled estimates (*P* < 0.0001, *P* = 0.01, respectively). It did not result in a statistically significant improvement of the 3-year SR (*P* = 0.07). There were fewer samples in the 3-year SR compared to the 1-year SR. This result is based on two small-sample trials. Additional samples are necessary to better understand the results. The combination therapy reduced the incidence of AEs including nausea and vomiting, fever, hepatalgia, increased transaminase, increased bilirubin and leukopenia (*P* < 0.00001). The result suggested that CKI increases the treatment efficacy and reduces AEs in HCC patients.

The combination therapy schedule is important in clinical practice. Therefore, CKI treatment duration was examined. In a subgroup analysis, the studies were divided into two durations: (1) the duration of the consecutive treatment of CKI exceeded 3 weeks in two trials (Han et al., 2012; Tian, 2014); (2) the duration of the remaining trials were approximately 2–3 weeks. The schedule choice did not result in differences in TR. The former schedule provided limited information regarding AEs (one trial’s report). The safety of CKI treatment for greater than 3 weeks should be further examined.

CKI suppressed the growth of various liver cancer cells such as H22 and HepG2 (Sun et al., 2011; Wang et al., 2011). The anti-tumor mechanisms of CKI may be involved in (1) the increased protein expression of p16, (2) the reduced unmethylated state of the p16 gene, (3) the inhibition of VEGF and MVD expression in neoplastic tissues, and (4) decreased microvessel density (Wang et al., 2015). Matrine, one of the major alkaloids in CKI, reduces cancer cell proliferation and induces apoptosis (Ma et al., 2008; Zhang et al., 2012). CKI is believed to be a complementary agent to TACE treatment to inhibit the growth of liver cancer, suppress tumor metastasis and improve the quality of life for patients with unresectable HCC.

Extensive research and strict methodologies were employed to select trials and analyze the efficacy and the AEs associated with HCC treatment. However, there are limitations to this research, such as the quality of the included data for the original trials. Many RCTs do not employ strict methodologies. Additionally, the majority of the studies examined Chinese patients. It is necessary to examine the results using a more varied population sample. The sample size, selection criteria, and TACE drugs varied for the included studies. We were unable to perform a subgroup analysis.

## Conclusion

These findings indicate that TACE combined with CKI may significantly improve TR, KPS and CP improvement and the 1- and 2-year SR. It is likely that the combination treatment also improves the 3-year SR, but the results were not statistically significant. The combination therapy was also associated with fewer AEs. However, our findings must be interpreted with caution because of the small sample size and limitations of the study. Several rigorous, large-scale RCTs are necessary to confirm these results.

## Author Contributions

XM, R-SL and Y-QH performed the search and wrote the manuscript. P-YL, JW, H-BS, R-LW analyzed the data. Y-MZ, H-HL, C-EZ and Z-JM performed the data extraction. JW and J-BW amended the paper. Y-LZ and X-HX designed the study and amended the paper.

## Conflict of Interest Statement

The authors declare that the research was conducted in the absence of any commercial or financial relationships that could be construed as a potential conflict of interest.

## Abbreviations

5-FU: fluorouracil
ADM: adriamycin
AEs: adverse events
CBM: Chinese Biomedical Database
CF: calcium folinate
CKI: Compound Kushen Injection
CI: confidence interval
CNKI: China National Knowledge Infrastructure
CP: Child-Pugh
DDP: cisplatin
EPI: epirubicin
GS: gelatin sponge
HCC: hepatocellular carcinoma
HCPT: hydroxycamptothecin
KPS: Karnofsky Performance Scale
M-H: Mantel-Haenszel
MMC: mitomycin
NR: not report
OR: odds ratio
RCTs: randomized clinical trials
SR: survival rate
SE: standard error
TACE: transarterial chemoembolization
THP: pirarubicin
TR: tumor response
VMIS: VIP medicine information system
