# Supplementary material for: The Therapeutic Efficacy and Safety of Compound Kushen Injection Combined with Transarterial Chemoembolization in Unresectable Hepatocellular Carcinoma: An Update Systematic Review and Meta-Analysis
Source: Front Pharmacol. 2016 Mar 31;7:70. doi: 10.3389/fphar.2016.00070 (PMC4814457; doi:10.3389/fphar.2016.00070)
Supplement: Supplementary file 1 [file Data_Sheet_1.DOC]

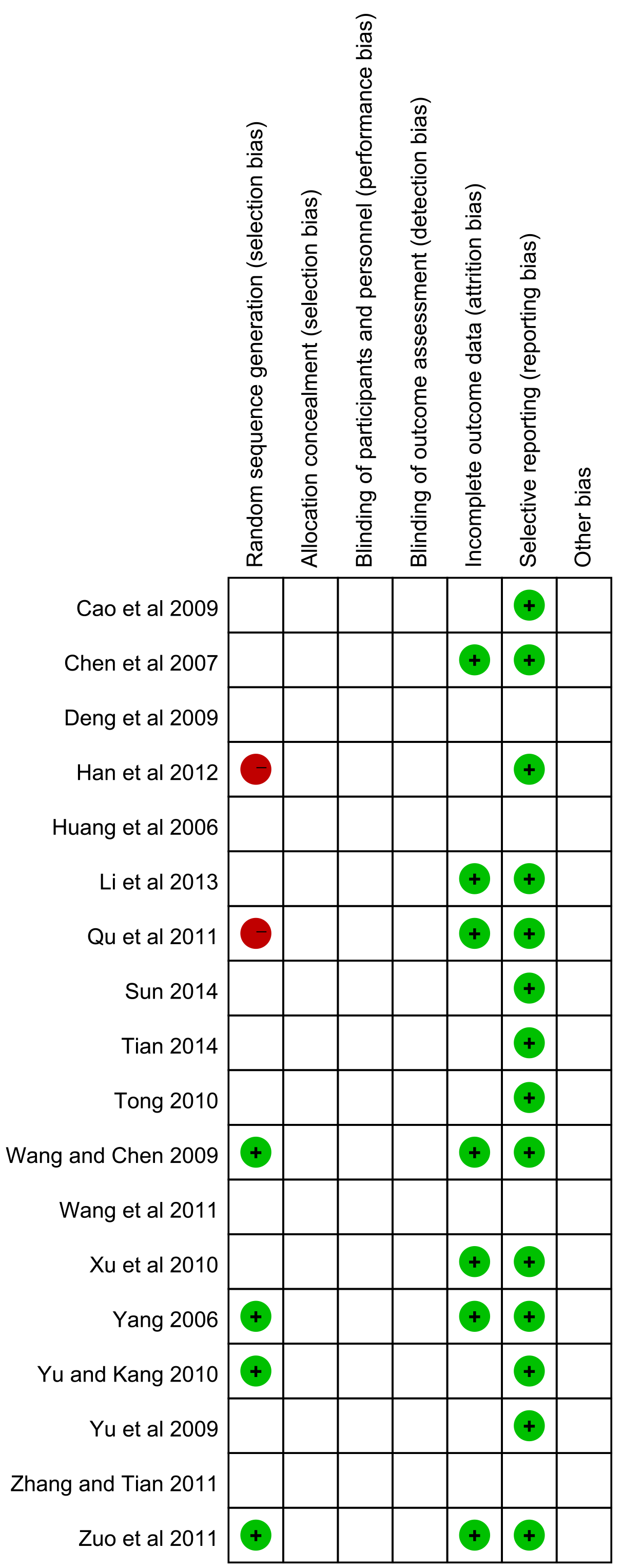


**Figure S1 Risk of bias summary of included trials.** Quality assessment was conducted by Review Manager 5.3 according to Cochrane Handbook for Systematic Reviews of Interventions Version 5.1.0. Red circle, high risk of bias; green circle, low risk of bias; blank, unclear risk of bias.


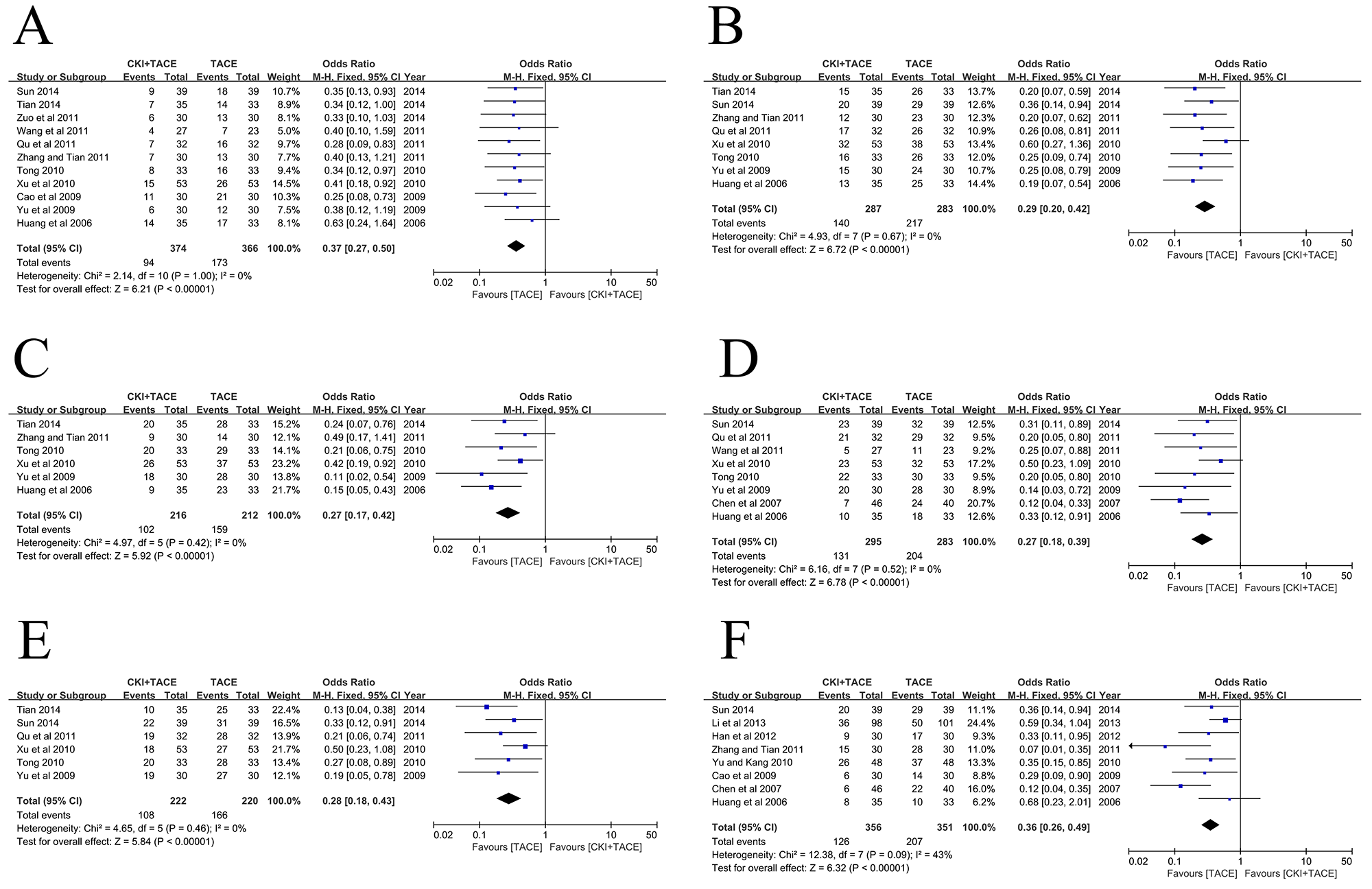


**Figure S2 Forest plot of adverse events in patients treated with CKI+TACE therapy and TACE alone.** (**A**) Forest plot of Nausea and vomiting rate; (**B**) Forest plot of fever rate; (**C**) Forest plot of hepatalgia rate; (**D**) Forest plot of increased transaminase rate; (**E**) Forest plot of increased bilirubin rate; (**F**) Forest plot of leukopenia rate. *I*^2^ and *P* is the criterion of heterogeneity test, ◆ pooled odds ratio, —■— odds ratio and 95% CI.

**Table S1 Outcome measures of included trials**

| Outcome measures | Efficacy | | Description |
| --- | --- | --- | --- |
| Tumor response^1^  (CR+PR) | CR | CT and/or MRI revealed complete clearance of the lesion. | |
|  | PR | Lesion decreased more than 50%. | |
|  | NC | Lesion decreased less than 50% or increased less than 25%. | |
|  | PD | Size of lesion increased more than 25% after treatment. | |
| KPS improvement^2^  (Improvement) | Improvement | KPS improved ≥ 10 points after treatment. | |
|  | Stabilization | KPS improved < 10 points or decreased < 10 points. | |
|  | Deterioration | KPS decreased ≥10 points after treatment. | |
| Child-Pugh improvement^3^  (Improvement) | Improvement | Child-Pugh improved ≥ 1 degree. | |
|  | Stabilization | Child-Pugh did not improve or decrease. | |
|  | Deterioration | Child-Pugh decreased ≥ 1 degree. | |

CKI, Compound Kushen Injection; TACE, Transarterial chemoembolization; KPS, Karnofsky Performance Scale. CR, complete response; PR, partial response; NC, no change; PD, progressive disease.

**Table S2 Baseline characteristics of the included** trials

| Study  (years) | Treatment  regimen | Number of Patients | Age (years)  Range,mean | Male/Female | Stage | KPS |
| --- | --- | --- | --- | --- | --- | --- |
| Tian, 2014 | CKI+TACE | 35 | 40-67,56.2 | 39/29 | II, III, IV | NR |
|  | TACE | 33 |  |  |  |  |
| Sun, 2014 | CKI+TACE | 39 | 48.7 | 23/16 | BCLC (B,C) | ≥60 |
|  | TACE | 39 | 51.5 | 27/12 |  |  |
| Li et al., 2013 | CKI+TACE | 98 | 35-67,59.7 | 139/60 | NR | ≥70 |
|  | TACE | 101 |  |  |  |  |
| Han et al., 2012 | CKI+TACE | 30 | 42-69,56 | 19/11 | II, III, IV | NR |
|  | TACE | 30 | 43-68,57 | 18/12 |  |  |
| Zuo et al., 2011 | CKI+TACE | 30 | 44.9 | 19/11 | BCLC (B) | NR |
|  | TACE | 30 | 44.0 | 21/9 |  |  |
| Qu et al., 2011 | CKI+TACE | 32 | 53 | 21/11 | BCLC (B,C) | ≥60 |
|  | TACE | 32 | 51 | 19/13 |  |  |
| Wang et al., 2011 | CKI+TACE | 27 | 26-61,43.2 | 32/18 | III, IV | ≥60 |
|  | TACE | 23 |  |  |  |  |
| Zhang and Tian, 2011 | CKI+TACE | 30 | 42-69,56.4 | 21/9 | II, III, IV | ≥60 |
|  | TACE | 30 | 45-67,57.9 | 19/11 |  |  |
| Xu et al., 2010 | CKI+TACE | 53 | 36-78,54 | 76/30 | II, III | NR |
|  | TACE | 53 |  |  |  |  |
| Tong, 2010 | CKI+TACE | 33 | 33-78,58 | 23/10 | III, IV | ≥60 |
|  | TACE | 33 | 32-79,57 | 24/9 |  |  |
| Yu and Kang, 2010 | CKI+TACE | 48 | 38-69,52.6 | 36/12 | II, III | ≥60 |
|  | TACE | 48 | 30-67,53.5 | 32/16 |  |  |
| Cao et al., 2009 | CKI+TACE | 30 | 37-84,61 | 36/24 | III, IV | ≥60 |
|  | TACE | 30 |  |  |  |  |
| Yu et al., 2009 | CKI+TACE | 30 | 33-78 | 20/10 | II, III, IV | NR |
|  | TACE | 30 | 32-79 | 22/8 |  |  |
| Deng et al., 2009 | CKI+TACE | 20 | 28-65,48 | 17/3 | III, IV | ≥70 |
|  | TACE | 20 | 26-66,53 | 18/2 |  |  |
| Wang and Chen, 2009 | CKI+TACE | 27 | 30-65,48 | 24/3 | II, III | ≥70 |
|  | TACE | 30 |  | 26/4 |  |  |
| Chen et al., 2007 | CKI+TACE | 46 | 40-71 | 62/24 | II, III | ≥70 |
|  | TACE | 40 |  |  |  |  |
| Yang, 2006 | CKI+TACE | 33 | 26-65,45.0 | 26/7 | II, III | ≥60 |
|  | TACE | 27 | 25-62,44.5 | 22/5 |  |  |
| Huang et al., 2006 | CKI+TACE | 35 | 35-72,53.5 | 25/10 | II, III | ≥50 |
|  | TACE | 33 | 32-74,53.0 | 24/9 |  |  |

CKI, Compound Kushen Injection; TACE, Transarterial chemoembolization; KPS, Karnofsky Performance Scale; NR, not report.

**References:**

1. Therasse, P. (2002). European Organisation for Research and Treatment of Cancer Data Center. Evaluation of response: new and standard criteria. *Ann Oncol*. Suppl 4, 127-129.
2. Yates, J. W., Chalmer, B., McKegney, F. P. (1980). Evaluation of patients with advanced cancer using the Karnofsky performance status. *Cancer*. 45(8),2220-2224.
3. Chen, H. Z. (2004). Practical internal medicine. Beijing: People's Medical Publishing House. The eleventh edition, 1856.
